# Understanding How Grammatical Aspect Influences Legal Judgment

**DOI:** 10.1371/journal.pone.0141181

**Published:** 2015-10-23

**Authors:** Andrew M. Sherrill, Anita Eerland, Rolf A. Zwaan, Joseph P. Magliano

**Affiliations:** 1 Department of Psychology, Northern Illinois University, DeKalb, Illinois, United States of America; 2 Department of Languages, Literature, and Communication, Utrecht University, Utrecht, The Netherlands; 3 Psychology Department, Erasmus University Rotterdam, Rotterdam, The Netherlands; The University of Nottingham, UNITED KINGDOM

## Abstract

Recent evidence suggests that grammatical aspect can bias how individuals perceive criminal intentionality during discourse comprehension. Given that criminal intentionality is a common criterion for legal definitions (e.g., first-degree murder), the present study explored whether grammatical aspect may also impact legal judgments. In a series of four experiments participants were provided with a legal definition and a description of a crime in which the grammatical aspect of provocation and murder events were manipulated. Participants were asked to make a decision (first- vs. second-degree murder) and then indicate factors that impacted their decision. Findings suggest that legal judgments can be affected by grammatical aspect but the most robust effects were limited to temporal dynamics (i.e., imperfective aspect results in more murder actions than perfective aspect), which may in turn influence other representational systems (i.e., number of murder actions positively predicts perceived intentionality). In addition, findings demonstrate that the influence of grammatical aspect on situation model construction and evaluation is dependent upon the larger linguistic and semantic context. Together, the results suggest grammatical aspect has indirect influences on legal judgments to the extent that variability in aspect changes the features of the situation model that align with criteria for making legal judgments.

## Introduction

Jurors are presented with at least two pieces of information that are the basis of the decision they make regarding the guilt of the defendant. The first includes a legal definition of a crime that was purportedly committed, which typically sets the constraints for making a legal decision about the case. The second includes descriptions of events that are to be considered by the jury with respect to whether or not a crime was committed. While many factors contribute to a final verdict, one should not overlook that the information gathered by jurors is often in the form of discourse. Recent evidence suggests that grammatical morphemes (e.g., tense, grammatical aspect) can bias how individuals mentally represent criminal activity and thereby possibly influence perceptions of the intentionality of a criminal action [1]. If so, grammatical morphemes could impact the perceived presence of dimensions specified in a legal definition (e.g., criminal intentionality), thus potentially impacting legal decision-making. The objective of the current study is to investigate how grammatical morphemes, and in particular grammatical aspect (defined below), may influence legal judgments.

Why would features of language matter in legal decision-making? Language can be viewed as a complex set of cues that help the construction of *situation models*, which are mental representations of the state of affairs being described in a text [2–4]. They reflect an understanding of how the described events are related along a number of dimensions such as space, time, and causality [4]. Moreover, when events involve people performing intentional actions, situation models capture how those actions are related to explicitly stated and inferred goals [5]. A situation model of a witness’s testimony should reflect many features of the event’s representation including agents (e.g., defendant and victim), instruments (e.g., gun), actions (e.g., pulling out gun), goals (e.g., to kill the victim), spatial arrangements (e.g., defendant running behind victim), and temporal information (e.g., gunshots before victim falls to ground) [3, 4]. While the words and combinations of words used in a text matter for situation model construction (e.g., [6, 7]), the grammatical and morphological features such as aspect may also play a role in conveying how events take place in the referenced situation [8–11].

Grammatical aspect is of particular interest in the present study. It is a morphological device used to convey information about the time course and duration of an activity [12–14]. The current paper examines two different aspectual categories: (a) the *imperfective* aspect in the past tense, which, in the progressive (vs. habitual) form, conveys an incomplete activity (e.g., was walking), and (b) the *perfective* aspect in the past tense, which conveys a completed activity (e.g., walked). In the case of the imperfective, an event is described as if seen endogenously as it unfolds, which can be described as “zooming into the event” [14, 15]. One assumption is that events described in the imperfective (vs. perfective) aspect are interpreted as being pertinent to the subsequent discourse and lead to the activation of more relevant event knowledge [9, 16, 17]. In other words, in addition to emphasizing the duration or time course of an event, the imperfective aspect may also function as a processing cue to emphasize and remember the given event and its constituent “internal” components (e.g., location, temporal dynamics, intentions of agents, instruments used). In contrast to the imperfective, the perfective describes an event as if seen exogenously as a whole, which can be described as “stepping back from the event” [14, 15].

The perfective aspect and imperfective aspect are often not interchangeable, particularly when each form conveys different information. For example, if one cannot complete a car trip to the beach due to a flat tire, one can say, “I was driving to the beach,” but one cannot say, “I drove to the beach.” In the first case, the imperfective, the event is described as unfolding, which allows for the possibility that it does not reach completion. In the second case, the perfective, the event is described as completed (i.e., one arrived at the beach).

Prior research has demonstrated that grammatical aspect can influence the form and structure of situation models in several ways [13, 18]. The imperfective aspect, when compared to the perfective aspect, more strongly emphasizes a depicted event’s temporal qualities [9, 19], locations [17], instruments [20], agents [21], and agent movement [10, 22–25]. Temporal dynamics are strongly linked to aspect choice [13]. For example, events described with an imperfective aspect are more likely to be perceived to have a longer duration than events described with a perfective aspect [9, 19].

Recent evidence suggests aspect can even have systematic and predictable influences on abstract representational systems such as perceiving intentionality in mundane and criminal behavior [1], as well as complex decision-making such as solving insight problems [26], voting [27], and evaluating résumés [28]. Of relevance to the present study, Hart and Albarracín presented participants with a description of an individual committing a violent crime [1]. The actions of the defendant were described with either an imperfective (e.g., *was firing* gun shots) or perfective aspect (e.g., *fired* gun shots). Participants judged the action to be more intentional when described in the imperfective aspect than the perfective aspect, which suggests variations to aspect can influence a juror’s legal judgments given that an explicit decision-making factor for jurors is the identification of criminal intentionality (i.e., *mens rea*) [29].

The goal of the present study was to assess the impact of grammatical aspect on legal decision-making. One limitation of Hart and Albarracín’s study is that participants were asked to make judgments about intentionality of the action but not asked to commit to a legal decision [1]. As noted in the opening paragraph, jurors are asked to apply a legal definition to the events of the case. As such, all experiments conducted in this study involve providing a legal definition and event description of a crime and then requiring participants to commit to a decision. Another limitation of Hart and Albarracín’s study is that the scenario they used ambiguously described the circumstances that potentially provoked the gunshots being fired (“After an argument broke out…”, p. 264). The ambiguity of the provocation in Hart and Albarracín’s scenario may have minimized considerations of how the perceived intentionality of the defendant may be linked to details of the provocation. To overcome these limitations, the first three experiments of the present study adopted scenarios that explicitly described a highly provocative event and a murder event. The fourth experiment, which is viewed as a conceptual replication of Hart and Albarracín’s study, includes minimal provocation.

## Experiment 1

In Experiment 1, participants read a description of a crime involving two agents, specifically the provocateur and the murderer. The grammatical aspect of the provocation and murder actions was varied such that they were described with either an imperfective aspect or perfective aspect. Participants were given a definition of first-degree murder that emphasized the importance of homicidal intentionality being operative prior to the action(s) that lead to the murder. Participants were asked to judge whether the murderer should be convicted of first- or second-degree murder. They also made judgments regarding the perceived intentionality of the murder, which is a critical definitional dimension within the legal definition of first-degree murder.

The materials used in Experiment 1 were designed to reflect real world murder scenarios that include two feuding agents. Thus, our scenarios contained provocation from the victim. The now infamous *Florida v*. *George Zimmerman* case is demonstrative of the importance of provocation in a murder trial. Interestingly, in his interview with the police, Zimmerman claimed he acted in self-defense and described the actions of the victim, Trayvon Martin, in an imperfective aspect (e.g., “He was wailing on my head […] and started hitting me into the sidewalk […]. I thought he was going for my firearm”). In contrast, Zimmerman described his own actions in a perfective aspect (e.g., “I just pulled out my firearm and shot him”) [30]. In such cases, the defendant may be motivated to more strongly emphasize the provocateur’s actions than his or her response. We anticipated that an emphasis on provocation could plausibly diminish other emphases on retaliatory actions. Manipulation of aspect in the provocation of the murder in our materials was exploratory and, therefore, we did not make specific predictions regarding its impact on first-degree murder decisions.

Experiment 1 afforded an assessment of an *intentionality hypothesis* with respect to how aspect may affect legal judgments. This hypothesis assumes the imperfective (vs. perfective) aspect cues the activation of intentionality, which should affect how participants reason about the application of the definition of first-degree murder to the scenario. As such, this hypothesis predicts participants will judge the murder to be (a) more intentional and (b) more reflective of first-degree murder when in the imperfective condition than the perfective condition. Further, perceived murderer intentionality will mediate a positive relationship between the murder in the imperfective (vs. perfective) aspect and the likelihood of first-degree murder judgments. In the current study, mediational analyses are necessary to assess if the hypothesized mechanisms (e.g., perceived intentionality) are indeed conduits through which aspect manipulations influence legal judgments (e.g., first-degree murder). Mediational analyses were only conducted when there was evidence that the manipulation of aspect affected perceptions of potential underlying mechanisms (e.g., perceived intentionality).

The inclusion of a provocation aspect manipulation was largely exploratory. However, one could expect that the murder would be perceived as especially justified if the provocation is perceived as severe. Given that actions described with a imperfective aspect are perceived to be more durative than those that are described with a perfective aspect [9, 19], it is reasonable to expect that first-degree murder judgments would be less likely when the provocation was conveyed with an imperfective aspect.

### Method

For the first and all following experiments, we report how we determined our sample size, all data exclusions, all manipulations, and all measures [31]. All materials and data for each experiment are publicly available on the Open Science Framework (URL: osf.io/vtka6). The institutional review board of the first and forth authors (Northern Illinois University) approved all methods across all four experiments. Written informed consent (agree/disagree checkbox) for each experiment was obtained from each participant.

#### Participants

The smallest effect size estimate from a previous study (Cohen’s *d* = .66; [1]) indicates a sample of at least 120 participants to detect a between-subjects effect of aspect on intentionality. In total, 140 participants were recruited from Amazon Mechanical Turk (M-Turk). All participants were located in the United States and were at least 18 years old. Participation required approximately four minutes. Compensation was nominal (US$0.10). Participants were excluded if they specified that English was not their first language, if participation time was less than two minutes, or if they did not conduct the experiment in a quiet environment with minimal distractions. The final sample consisted of 123 participants (see Table 1 for sample characteristics of all four experiments).

**Table 1:** Sample Characteristics and Random Assignment for Each Experiment.

				Experiment Number			
				*1*	*2*	*3*	*4*
**Excluded Participants**	***N***			**17**	**16**	**35**	**20**
	***Exclusionary Criteria***		Short duration (< 2 minutes)	8	8	3	2
			Noise and/or many distractions	5	2	5	2
			Non-native English speakers	4	6	27	16
**Included Participants**	***N***			**123**	**130**	**307**	**139**
	***Demographics***	*Age*	Mean	33.9	31.3	32.3	32.4
			Standard Deviation	12.6	10.8	11.3	10.4
		*Gender (%)*	Male	48.8	54.6	52.4	56.8
			Female	51.2	45.4	47.6	43.2
		*Racial Identity (%)*	White or European American	85.4	83.8	85.3	83.5
			Asian or Asian American	8.1	8.5	3.9	7.9
			Black or African American	6.5	3.8	6.5	6.5
			Pacific Islander	0	0	0.3	0
			Native American	0	1.5	1.0	0.7
			Multiracial / Other	0	2.3	2.9	1.4
		*Hispanic*	Yes	6.5	3.8	8.1	10.1
		*Heritage (%)*	No	93.5	96.2	91.9	89.9
		*Education*	Less than high school diploma	0	1.5	0.3	0
		*Level (%)*	High school diploma	17.9	12.3	12.7	12.2
			Some college	28.5	28.5	30.0	33.1
			Associate’s degree	11.4	11.5	12.1	7.9
			Bachelor’s degree	34.1	33.1	37.1	37.4
			Graduate degree	8.1	13.1	7.8	9.4
		*Employment Status (%)*	More than 35 hours per week	32.5	49.2	40.1	50.4
			Less than 35 hours per week	37.4	23.8	28.7	24.5
			Not employed, looking for work	14.6	13.8	13.0	9.4
			Not employed, not looking	9.8	9.2	12.1	12.2
			Retired	4.1	2.3	2.3	0.7
			Disabled and not able to work	1.6	1.5	3.6	2.9
	***Random Assignment (%)***	Murder: *Perfective*	Provocation: *perfective*	24.4	25.4	28.3	0
			Provocation: *imperfective*	22.8	25.4	27.0	0
			Minimal provocation	0	0	0	55.4
		Murder: *Imperfective*	Provocation: *perfective*	25.2	24.6	21.2	0
			Provocation: *imperfective*	27.7	24.6	23.5	0
			Minimal provocation	0	0	0	44.6

#### Materials and Procedures

**Fig 1:**
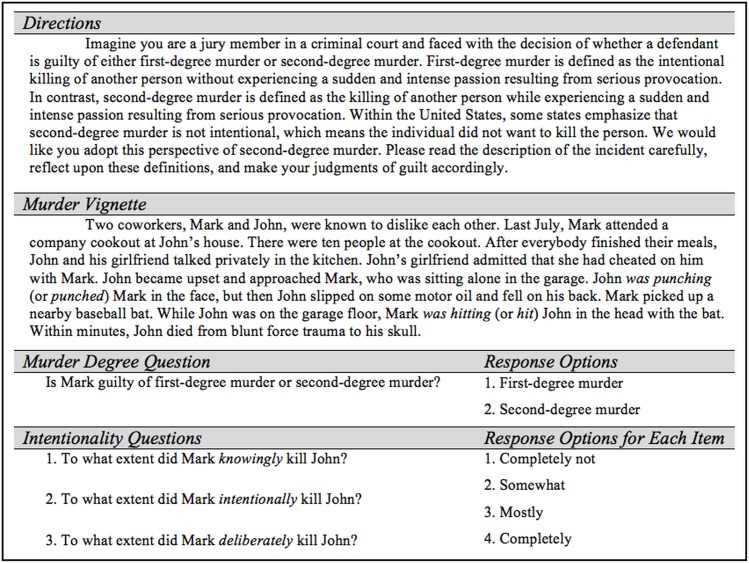
Materials used in Experiment 1.

The materials consisted of directions, a murder vignette, and a set of questions about the murder (see Fig 1), which were viewed on separate webpages that could not be re-visited after pressing the “next” button. Participants were asked to take the perspective of a juror in a murder trial. The directions contain definitions of first- and second-degree murder based on Illinois law [32]. The convictions are distinguished by intentionality (first-degree murder) versus “sudden and intense passion resulting from serious provocation” (second-degree murder). The murder vignette described a set of circumstances that led to the provocation and murder actions. The provocation and murder actions both involved a punctual verb (punch, hit). Participants were randomly assigned to read one of four versions of the vignette. Participants were asked to make a conviction (i.e., first- or second-degree murder) and to answer three intentionality questions that combine into a composite score (adapted from [1]). Next, participants completed a brief questionnaire assessing gender, age, native language, race, Hispanic heritage, education level, employment status, and the extent to which their current environment included distractions and noise. Lastly, participants were asked about their views of the death penalty and to guess the purpose of the study; these items were included for exploratory purposes and the results are not discussed in this paper.

### Results and Discussion

First, did participants use the legal definition during reasoning? As expected given the definition of first-degree murder, judgments of first-degree murder were significantly and positively correlated with judgments of intentionality, *r*(121) = .53, *p* < .001.

Second, did aspect manipulations affect perceptions of the presence of murderer intentionality, which was the critical dimension specified in the legal definition? The three intentionality items were strongly interrelated (α = .90) and combined using mean scores. The mean intentionality score in the sample was 2.63 (*SD* = 0.90). Using a 2 (Provocation Aspect) X 2 (Murder Aspect) ANOVA on intentionality judgments, no significant effects were found for the provocation aspect, *F*(1, 119) = 2.39, *p* = .125, η^2^ = .020, murder aspect, *F*(1, 119) = 0.99, *p* = .322, η^2^ = .008, or the interaction term, *F*(1, 119) = 0.02, *p* = .892, η^2^ < .001. Thus, the aspectual manipulation did not influence perceived intentionally. The criterion was not met to test for a mediating role of perceived intentionality on a possible relationship between aspect manipulations and legal judgments.

Third, did aspect manipulations affect legal judgments? In order to test the extent that aspect of the murder and provocation affected legal judgments, a binary logistic regression analysis was conducted to predict first-degree murder judgments (1 = first-degree murder, 0 = second-degree murder) using as predictors the aspectual categories of the provocation verb (perfective = 1, imperfective = 0) and the murder verb (perfective = 1, imperfective = 0), as well as an interaction term. The full model was statistically significant, χ^2^ = 8.87, *p* = .031, *Nagelkerke’s R* ^*2*^ = .096. The Wald criterion demonstrated a main effect of the provocation aspect (*p* = .038), wherein reading provocation in the perfective (vs. imperfective) increased the likelihood of a first-degree murder judgment by 3.176 times. There was no main effect of the murder verb (*p* = .680) or interaction effect (*p* = .943).

In sum, the results of Experiment 1 did not support the intentionality hypothesis. However, the significant main effect of the grammatical aspect of the provocation action on first-degree murder judgments suggests that aspect can affect legal judgments but not based on the hypothesized reasons. As noted in the introduction, it is well established that grammatical aspect affects how individuals understand the temporal dynamics of an event [9, 19]. It may be the case that impact of grammatical aspect on legal decision-making may be constrained to understanding the temporality of the events. That is, provocation that is conveyed in an imperfective aspect may be perceived as more durative, and therefore more severe.

## Experiment 2

The purpose of Experiment 2 was to replicate Experiment 1 in order to reexamine the null effect associated with the intentionality hypothesis. Additionally, given the preponderance of studies showing that aspect affects the perceived temporal feature of events, we queried participants about the number of iterations of the provocation and murder actions. Iterations were specified rather than duration because the verbs depicted punctual events (i.e., actions that end almost at the same time they begin). In this situation, imperfective aspect implies the event is iterative (e.g., hit many times as opposed to one instance). Moreover, as can be seen in Fig 1, the legal definition was not restricted to intentionality and specified individuals should consider the severity of the provocation, evidence of self-defense, and the amount of sudden passion felt by the murderer. As such, questions were added regarding these dimensions in order to evaluate if the definition was used and if the manipulation of aspect affected these dimensions.

A *temporal perspective hypothesis* assumes that aspect affects the perceived number of iterations of the provocation or murder event such that the number of iterations would be judged to be higher in the imperfective condition than in the perfective condition. As was the case with Experiment 1, if there were direct effects of aspect manipulations on perceptions of situational features (e.g., provocation iteration, intentionality), mediational analyses were planned to assess indirect effects of aspect on legal judgments through these potential underlying mechanisms.

### Method

#### Participants

In total, 146 participants were recruited from M-Turk, all located in the United States and at least 18 years old. Compensation for participation (approximately four minutes) was nominal (US$0.25). Using the same exclusionary criteria as Experiment 1, the final sample consisted of 130 participants (see Table 1).

#### Materials and Procedures

**Fig 2:**
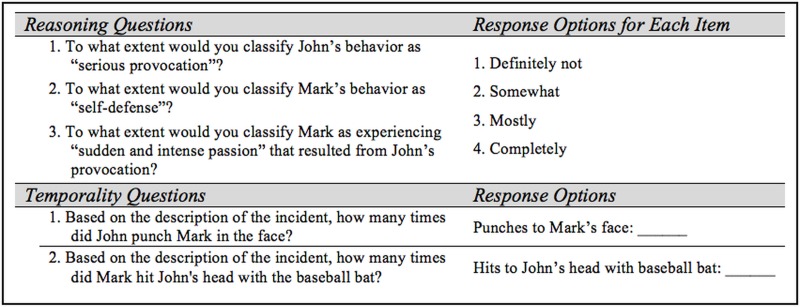
Additional materials used in Experiment 2.

The vignette was identical to that of Experiment 1 with the exception of the last sentence. “Within minutes, John died from blunt force trauma to his skull” was changed to “As a result, John died.” This change was intended to remove potential confusion because in the original version it was unclear if the murder action(s) occurred for a duration of minutes or if minutes passed after the murder action(s). Additionally, questions were added to assess the extent participants felt various dimensions specified in the definition were present in the scenario, as well as perceived temporality of the murder and provocation (see Fig 2). All procedures were the same as Experiment 1.

### Results and Discussion

First, did participants use the legal definition during reasoning? As can be seen in Table 2, all dimensions specified in the definition were significantly correlated with legal judgments and in the expected direction. Specifically, perceived intentionality of the murder (α = .91) was positively correlated with decisions of first-degree murder, whereas seriousness of provocation, self-defense, and sudden passion were negatively correlated with that decision.

**Table 2:** Correlations from Experiment 2.

Measures	*2*	*3*	*4*	*5*	*6*	*7*	*M (SD)*
**1. Legal Judgment**	.611**	-.096	-.099	-.450**	-.312**	-.606**	0.38 (0.49)
**2. Intentionality (Murderer)**	*–*	-.026	.047	-.275**	-.286**	-.332**	2.37 (0.94)
**3. Iterations (Provocateur)**		*–*	.253**	.173*	.113	.049	2.55 (2.36)
**4. Iterations (Murderer)**			*–*	-.126	-.073	.040	3.12 (2.85)
**5. Serious provocation**				*–*	.298**	.571**	2.76 (1.03)
**6. Self-defense**					*–*	.240**	1.61 (0.78)
**7. Sudden Passion**						*–*	3.13 (0.94)

Legal judgments: 0 = Second-degree murder, 1 = First-degree murder.

* *p* < .05.

** *p* < .01.

Second, did aspect manipulations affect perceptions of the presence of dimensions specified in the legal definition? A series of 2 (Provocation Aspect) X 2 (Murder Aspect) ANOVAs were conducted (see Table 3). With respect to intentionality judgments, there was a significant main effect of murder aspect, indicating higher judgments in the imperfective condition (*M* = 2.55, *SD* = 0.89) than the perfective condition (*M* = 2.20, *SD* = 0.96), *t*(128) = 2.15, *p* = .034. This finding provides support for the intentionality hypothesis. With respect to the other dimensions specified by the legal definition, there was a significant main effect of provocation aspect on judgments of the seriousness of the provocation, such that judgments were higher when provocation was described in the imperfective aspect (*M* = 2.97, *SD* = 1.00) than perfective aspect (*M* = 2.55, *SD* = 1.02), *t*(128) = 2.35, *p* = .020. With respect to temporality judgments, results support the temporal perspective hypothesis. Specifically, provocation iteration judgments were higher when provocation was described in the imperfective aspect (*M* = 3.92, *SD* = 2.64) than perfective aspect (*M* = 1.17, *SD* = 0.67), *t*(128) = 8.16, *p* < .001. Likewise, murder iteration judgments were higher when murder was described in the imperfective aspect (*M* = 4.83, *SD* = 2.96) than perfective aspect (*M* = 1.45, *SD* = 1.39), *t*(128) = 8.35, *p* < .001.

**Table 3:** Experiment 2 Group Means (Provocation Aspect X Murder Aspect) and ANOVA Results.

	Imperfective Provocation				Perfective Provocation				ANOVA Results		
	Imperfective Murder		Perfective Murder		Imperfective Murder		Perfective Murder		Provocation	Murder	Interaction
**Variable**	*M*	*SD*	*M*	*SD*	*M*	*SD*	*M*	*SD*	*F* (*η*_P_^2^)	*F* (*η*_P_^2^)	*F* (*η*_P_^2^)
Intentionality (Murderer)	2.40_a_	0.86	2.03_b_	0.77	2.70_a_	0.92	2.36_b_	1.11	3.86 (.030)†	4.68 (.036)*	0.01 (< .001)
Iterations (Provocateur)	4.31_a_	3.02	3.55_a_	2.18	1.23_b_	0.79	1.12_b_	0.55	67.16 (.348)***	1.65 (.013)	0.99 (.008)
Iterations (Murderer)	4.75_a_	2.98	1.76_b_	1.84	4.91_a_	2.99	1.15_b_	0.62	0.31 (.002)	69.23 (.355)***	0.88 (.007)
Serious Provocation	2.91_a_	1.06	3.03_a_	0.95	2.41_b_	0.98	2.70_b_	1.05	5.54 (.042)*	1.37 (.011)	0.22 (.002)
Self-Defense	1.72	0.73	1.73	0.80	1.41	0.50	1.58	1.00	2.87 (.022)†	0.42 (.003)	0.35 (.003)
Sudden Passion	3.31	0.78	3.13	0.98	3.00	1.02	3.09	0.98	1.10 (.009)	0.09 (.001)	0.70 (.006)

Means in the same row that do not share the same subscript differ from each other at the *p* < .05 level. Df for all ANOVAs is 126.

^†^ *p* < .10.

* *p* < .05.

*** *p* < .001.

Third, did aspect manipulations affect legal judgments? The same binary logistic regression conducted for Experiment 1 was conducted for Experiment 2. The full model showed a non-significant trend, χ^2^ = 6.46, *p* = .091, *Nagelkerke’s R* ^*2*^ = .066. Using the Wald criterion, the aspectual category of the provocation verb showed a non-significant trend (*p* = .076), wherein reading provocation in the perfective (vs. imperfective) increased the likelihood of a first-degree murder judgment by 2.556 times. The aspectual manipulation on the murder verb was again not significant (*p* = .939), nor was the interaction term (*p* = .981). Thus, this non-significant trend is consistent with the direction of results of Experiment 1.

Fourth, what are the relationships between aspect manipulations, perceptions of dimensions in the legal definition (i.e., perceived intentionality and serious provocation), and legal judgments? A mediational analysis assessed the intentionality hypothesis prediction that murder in the imperfective enhances murder intentionality and thus higher first-degree murder judgment rates as per the legal definition. Consistent with this prediction, a mediational analysis (10,000 bootstrap samples using PROCESS procedure) found that the murder aspect, when controlling for the provocation aspect, predicted murder-degree convictions indirectly through perceived intentionality. Specifically, when murder is presented in the imperfective (vs. perfective), participants perceived higher intentionality (*a* = -0.35, *p* = .032), which strongly predicts the likelihood of a first-degree conviction (*b* = 1.84, *p* < .001). The indirect link between the aspectual category of murder and murder-degree judgments was significant (*ab* = -0.64; 95% bias-corrected bootstrap CI [-1.35, -0.04]), while the direct link was not significant (*c*’ = 0.66, *p* = .176). Next, a mediational analysis assessed the temporal perspective hypothesis prediction that provocation in the imperfective enhances perception of serious provocation and thus reduces first-degree murder judgment rates as per the legal definition. Consistent with this prediction, the provocation aspect, when controlling for the murder aspect, predicted murder-degree convictions indirectly through perceived serious provocation. Specifically, when provocation is presented in the imperfective (vs. perfective), participants perceived more serious provocation (*a* = -0.42, *p* = .020), which strongly reduces the likelihood of a first-degree conviction (*b* = -1.01, *p* < .001). The indirect link was significant (*ab* = 0.42; 95% bias-corrected bootstrap CI [0.06, 0.92]), while the direct link showed a non-significant trend (*c*’ = -0.70, *p* = .091).

In sum, the results of Experiment 2 provide evidence that participants use legal definitions when making decisions and that grammatical aspect may influence this process by emphasizing representational systems such as temporal dynamics and perceived intentionality, thus supporting both the temporal perspective hypothesis and the intentionality hypothesis. The mediational analyses suggest the impact of aspect on legal judgments is indirect. That is, aspect affects how people reason to the extent that the affected scenario reflects the dimensions specified in the definition. Given that the temporal perspective hypothesis and the intentionality hypothesis are not mutually exclusive, support for each hypothesis does not contradict the other. In the given text, readers were asked to evaluate the inextricably linked behaviors of the provocateur and the murderer, so any impact of aspect on the representation of one agent could also impact the event as whole. However, the indirect effects (perceived serious provocation and intentionality) were by no means as robust as the direct impact of aspect on the perceived temporal dynamics of the events in the scenario. One possibility to explore is that the impact of the imperfective aspect on immediate representational systems, such as temporal dynamics, may have a serial effect on distal representational systems, such as perceived intentionality.

## Experiment 3

In Experiments 1 and 2, provocation was described prior to the murder. However, in real world discourse, scenarios can differ in semantic context and structure. The reported direct effect of provocation aspect on legal judgments may be a consequence of mentioning the provocation before the murder. There is evidence that first mentioned events have a special status in situation models [33, 34]. Experiment 3 was conducted to test the possibility that the influence of aspect may be contingent on various linguistic features embedded within a given text, namely order of mention. Thus, the experimental scenario of Experiment 3 was largely the same as the first two experiments but the murder was mentioned first and the provocation was mentioned second. Assuming the order of mention does indeed shift emphasis away from the provocation and toward the murder, we expected that murder in the imperfective (vs. perfective) would result in greater perceived intentionality and rates of first-degree murder decisions. Further, we planned to explore the question raised by Experiment 2 that the influence of aspect on murderer intentionality can be mediated through the perceived temporality of the murder action. This possibility assumes a serial multiple mediation effect whereby the imperfective (vs. perfective) aspect leads to a greater number of murder actions, which leads to greater perceived murder intentionality, which then leads to a higher likelihood of first-degree murder judgments.

### Method

#### Participants

Based on a power analysis conducted using the results of Experiments 1 and 2, we set a minimum sample size of 265. In total, 342 participants were recruited from M-Turk with 307 meeting inclusion criteria (see Table 1). All participants were at least 18 years old and located within the United States. Participation (approximately four minutes) was compensated nominally (US$0.25).

#### Materials and Procedures

**Fig 3:**
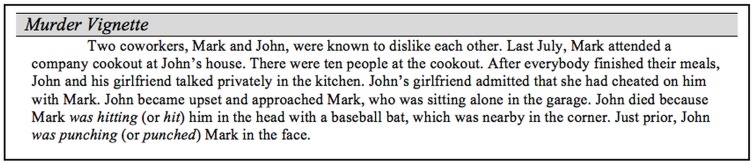
Vignette used in Experiment 3.

We altered the vignette used in Experiment 2 so that the order of mention was reversed and the detail about motor oil on the floor was removed (see Fig 3). In addition, three provocateur intentionality items that mirror murderer intentionality items were included for exploratory purposes. The procedure was the same as the previous experiments.

### Results and Discussion

First, did participants use the legal definition during reasoning? As can be seen in Table 4, the correlations are completely consistent with those from Experiment 2. Specifically, perceived intentionality of the murder was positively correlated with decisions of first-degree murder, whereas seriousness of provocation, self-defense, and sudden passion were negatively correlated with that decision.

**Table 4:** Correlations from Experiment 3.

Measures	*2*	*3*	*4*	*5*	*6*	*7*	*8*	*M (SD)*
**1. Legal Judgment**	-.006	.531**	.130*	.158**	-.234**	-.147*	-.340**	0.16 (0.37)
**2. Intentionality (Provocateur)**	–	.043	.075	.101	.078	.041	.172**	3.48 (0.73)
**3. Intentionality (Murderer)**		–	.100	.340**	-.160**	-.330**	-.217**	2.09 (0.87)
**4. Iterations (Provocateur)**			–	.246**	-.021	.075	.033	2.52 (2.79)
**5. Iterations (Murderer)**				–	-.069	-.199**	-.045	3.25 (3.76)
**6. Serious Provocation**					–	.380**	.369**	2.85 (0.93)
**7. Self-Defense**						–	.061	2.06 (1.03)
**8. Sudden Passion**							–	3.18 (0.91)

Legal judgments: 0 = Second-degree murder, 1 = First-degree murder.

* *p* < .05.

** *p* < .01.

Second, did aspect manipulations affect perceptions of the presence of temporal dynamics and the definitional dimensions? A series of 2 (Provocation Aspect) X 2 (Murder Aspect) ANOVAs were conducted on these outcomes (see Table 5). With respect to the intentionality judgments, there was again support for the intentionality hypothesis. Specifically, a main effect of murder aspect was found such that judgments of intentionality where higher when conveyed in imperfective (*M* = 2.28, *SD* = 0.88) than perfective (*M* = 1.94, *SD* = 0.84), *t*(305) = 3.52, *p* < .001. Further, there was a main effect of provocation aspect on self-defense and a non-significant trend of murder aspect on perceptions of serious provocation; due to a lack of a priori predictions, these findings will not be explored further. With regard to temporal dynamics, as with Experiment 2, ANOVA results on the temporal judgments were consistent with the temporal perspective hypothesis. There was a main effect of the provocation aspect on the perceived number of provocation iterations such that there were higher judgments in the imperfective condition (*M* = 3.98, *SD* = 3.24) than perfective condition (*M* = 1.03, *SD* = 0.81), *t*(305) = 10.92, *p* < .001. Likewise, there was a main effect of the murder aspect on the perceived number of murder iterations such that there were higher judgments in the imperfective aspect (*M* = 5.74, *SD* = 4.38) than perfective aspect (*M* = 1.25, *SD* = 1.09), *t*(305) = 12.88, *p* < .001.

**Table 5:** Experiment 3 Group Means (Provocation Aspect X Murder Aspect) and ANOVA Results.

	Imperfective Provocation				Perfective Provocation				ANOVA Results		
	Imperfective Murder		Perfective Murder		Imperfective Murder		Perfective Murder		Provocation	Murder	Interaction
**Variable**	*M*	*SD*	*M*	*SD*	*M*	*SD*	*M*	*SD*	*F* (*η*_P_^2^)	*F* (*η*_P_^2^)	*F* (*η*_P_^2^)
Intentionality (Provocateur)	3.48	0.67	3.56	0.69	3.53	0.71	3.39	0.82	0.50 (.002)	0.13 (< .001)	1.74 (.006)
Intentionality (Murderer)	2.28_a_	0.90	1.86_b_	0.81	2.29_a_	0.86	2.00_b_	0.87	0.58 (.002)	12.47 (.040)***	0.44 (.001)
Iterations (Provocateur)	4.61_a_	3.55	3.43_a_	2.85	1.11_b_	1.16	0.97_b_	0.39	123.34 (.289)***	6.02 (.019)*	3.71 (.012)†
Iterations (Murderer)	5.76_a_	4.77	1.47_b_	1.46	5.71_a_	3.94	1.05_b_	0.46	0.47 (.002)	164.40 (.352)***	0.28 (.001)
Serious Provocation	2.82	0.97	3.02	0.87	2.68	1.03	2.85	0.87	2.19 (.007)	3.14 (.010)†	0.02 (< .001)
Self-Defense	2.17_a_	1.06	2.27_a_	1.09	1.82_b_	0.97	1.97_b_	0.97	7.67 (.025)**	1.09 (.004)	0.05 (< .001)
Sudden Passion	3.08	1.04	3.32	0.80	3.17	0.96	3.14	0.86	0.20 (.001)	0.93 (.003)	1.59 (.005)

Means in the same row that do not share the same subscript differ from each other at the *p* < .05 level. Df for the following ANOVAs is 303: Intentionality (Murderer), Iterations (Provocateur), Iterations (Murderer), and Serious Provocation. Df for the following ANOVAs is 302: Intentionality (Provocateur), Self-Defense, and Sudden Passion.

^†^ *p* < .10.

* *p* < .05.

** *p* < .01.

*** *p* < .001.

Third, did aspect manipulations affect legal judgments? Using the same binary logistic regression analysis as the previous experiments, the full model was statistically non-significant, χ^2^ = 3.24, *p* = .356, *Nagelkerke’s R* ^*2*^ = .018. Thus, none of the predictors (provocation aspect, murder aspect, interaction term) had a statistically significant total effect on legal judgments. As such, we did not replicate the findings from Experiments 1 and 2, indicating that the temporal ordering of the provocation and murder actions affected the impact of aspect on legal judgments.

Fourth, what are the relationships between aspect manipulations, temporal dynamics, perception of dimensions in legal definitions (i.e., perceived intentionality), and legal judgments? Three mediational analyses were conducted: two simple mediation models that assess the temporal perspective hypothesis and intentionality hypothesis and a serial multiple mediation model that assesses the exploratory possibility that the influence of the imperfective aspect impacts temporal dynamics, which then impact perceived intentionality, which then impacts legal judgments.

The first mediation model was consistent with the temporal perspective hypothesis as applied to the murder action. Specifically, the mediational analysis found that the murder aspect, when controlling for the provocation aspect, predicted first-degree murder convictions indirectly through the perceived number of murder actions. When murder was presented in the imperfective (vs. perfective), participants perceived more murder actions (*a* = -4.47, *p* < .001), which was linked to the likelihood of a first-degree conviction (*b* = 0.12, *p* = .010). The indirect link between the aspectual category of murder and legal judgments was significant (*ab* = -0.51; 95% bias-corrected bootstrap CI [-0.93, -0.09]), while the direct link was not significant (*c*’ = 0.33, *p* = .413).

The second mediation model was consistent with the intentionality hypothesis as applied to the murder action. When controlling for the provocation aspect, the murder aspect predicted first-degree murder convictions indirectly through perceived intentionality. Specifically, when murder was presented in the imperfective (vs. perfective), participants perceived more intentionality (*a* = -0.35, *p* < .001), which was strongly linked to the likelihood of a first-degree conviction (*b* = 1.71, *p* < .001). The indirect link between the aspectual category of murder and legal judgments was significant (*ab* = -0.60; 95% bias-corrected bootstrap CI [-1.01, -0.24]), while the direct link was not significant (*c*’ = 0.31, *p* = .414).

**Fig 4:**
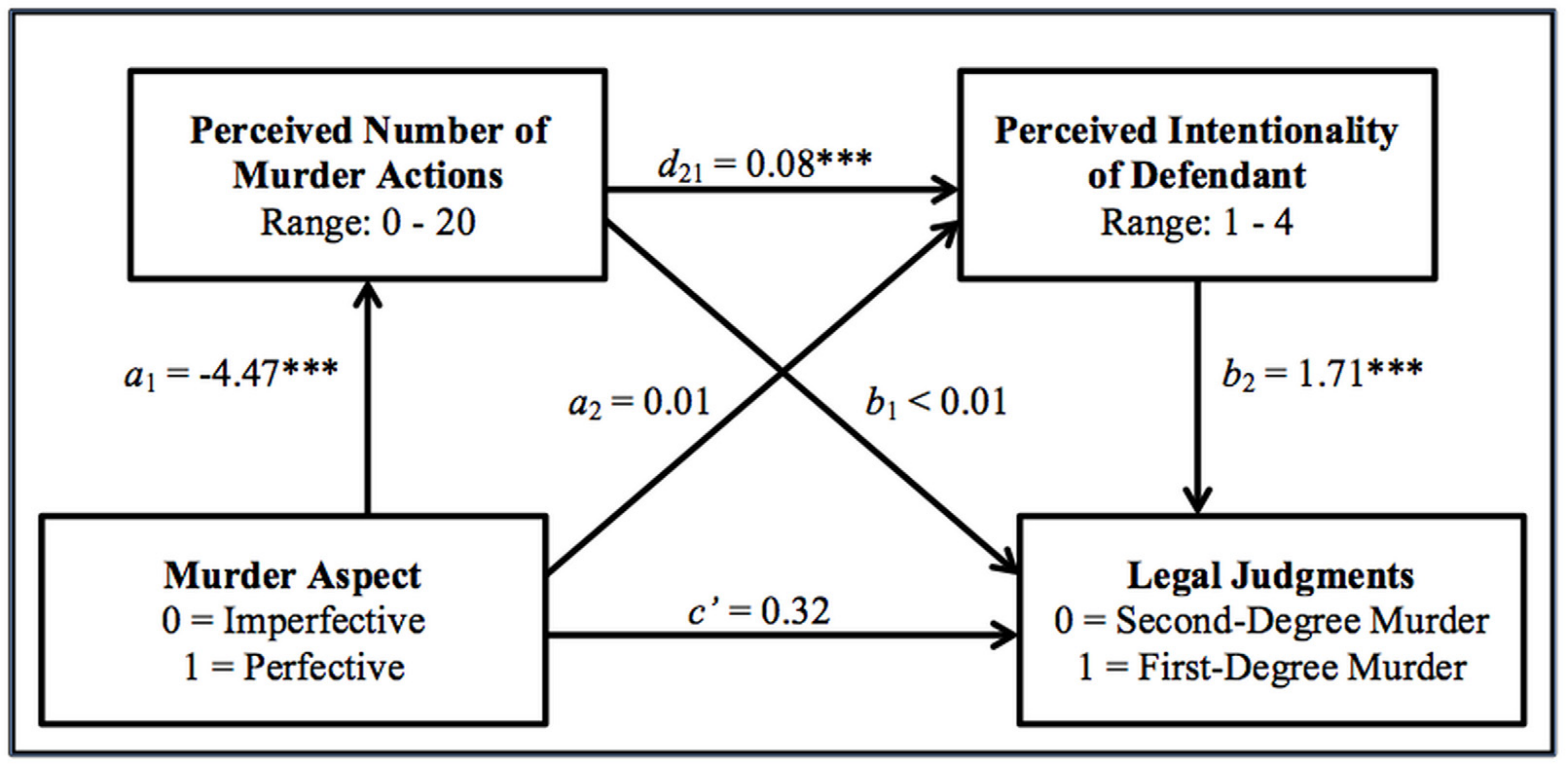
Schematic representation of the serial multiple mediational analysis. ***p < .001.

The third mediation model was consistent with the possibility that conveying the murder action in the imperfective (vs. perfective) leads to higher rates of first-degree murder judgments via both hypothesized mechanisms: perceived temporality and perceived intentionality. A serial multiple mediator analysis showed that the murder aspect, controlling for the provocation aspect, influenced legal judgments indirectly through perceived number of murder actions and then perceived intentionality. No support was found for the opposite serial order. Specifically, when murder was presented in the imperfective (vs. perfective), participants perceived higher iterations of murder actions (*a*_1_ = -4.47, *p* < .001), which then predicted perceptions of intentionality (*d*_21_ = 0.08, *p* < .001), which then finally predicted the likelihood of a first-degree conviction (*b*_2_ = 1.71, *p* < .001). The indirect link between the aspectual category of murder and legal judgments was significant (*a*_*1*_ *d*_*21*_ *b*_*2*_ = -0.62; 95% bias-corrected bootstrap CI [-0.96, -0.32]), while the direct link was not significant (*c*’ = 0.32, *p* = .497; see Fig 4).

In sum, the serial multiple mediational analysis demonstrates that aspect can indeed influence legal judgments but the effects are indirect. The aspect of the murder had a primary effect on temporal dynamics, which influenced perceived intentionality, which then impacted judgments. Thus, perceived iterations of the murder influenced the extent to which participants applied the legal definition to the given scenario. Further, results from Experiment 3 demonstrate that actions that are emphasized by multiple systems (e.g., first mention, imperfective aspect) appear to have the greatest impact on situation model construction and evaluation. This finding illustrates how the influence of aspect is constrained by other contextual features. In other words, many dynamic boundary conditions exist for the psychological impact of aspect. Texts may be arranged to allow aspectual manipulations to produce effects with a range of magnitudes. To demonstrate this possibility, Experiment 4 was designed to mirror the previous experiments with a notable change of removing physical provocation, thus depicting a minimally provoked murder scenario.

## Experiment 4

Experiment 4 was designed to demonstrate potential boundary conditions to the effects observed in the first three experiments. To achieve this aim, Experiment 4 used similar materials to the first three experiments with a notable reduction in provocation. The minimally provoked murder vignette is viewed as a conceptual replication of the materials used by Hart and Albarracín [1]. The intentionality hypothesis predicts murder in the imperfective will enhance perceived intentionality and, in turn, legal judgments. Following Experiment 3, we planned to explore a mediating role of murder temporality on the potential relationship between aspect and intentionality. However, it was also anticipated that the pattern of results from the first three experiments might not emerge because the nature of the situation model had been substantially changed due to the reduction of provocation. Any added emphasis via the imperfective aspect may be inconsequential if readers attend closely to the minimally provoked nature of the murderer’s action. That is, aspectual manipulations may not override the major semantic components of the situation (i.e., a minimally provoked murder).

### Method

#### Participants

Based on a power analysis conducted using the results of Experiments 1 and 2, we set a minimum sample size of 133. In total, 159 participants were recruited from M-Turk with 139 meeting inclusion criteria (see Table 1). All participants were at least 18 years old and located within the United States. Participation (approximately four minutes) was compensated nominally (US$0.25).

#### Materials and Procedures

**Fig 5:**
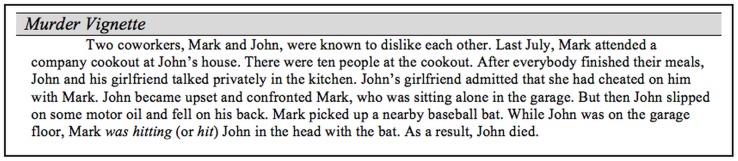
Vignette used in Experiment 4.

All materials were the same as Experiment 2; however, the vignette was altered so that no physical provocation was mentioned as the provocateur initially confronts the eventual murderer in the garage (see Fig 5). Accordingly, items assessing provocateur intentionality and provocation temporality were removed. The procedures were the same as the previous experiments.

### Results and Discussion

First, did participants use the legal definition during reasoning? The results are consistent with those of Experiments 2 and 3, indicating that the definition was used to make the legal judgments (see Table 6). Specifically, perceived intentionality of the murder was positively correlated with decisions of first-degree murder, whereas seriousness of provocation and sudden passion where negatively correlated with that decision. Judgments of self-defense were not significantly correlated with the first-degree murder decision but the coefficient is in a similar direction.

**Table 6:** Correlations from Experiment 4.

Measures	*2*	*3*	*4*	*5*	*6*	*M (SD)*
**1. Legal Judgment**	.674**	.128	-.419**	-.154	-.534**	0.54 (0.50)
**2. Intentionality (Murderer)**	–	.054	-.268**	-.178*	-.332**	3.01 (0.92)
**3. Iterations (Murderer)**		–	-.121	.020	-.017	3.19 (3.95)
**4. Serious Provocation**			–	.319**	.529**	2.18 (0.95)
**5. Self-Defense**				–	.204*	1.27 (0.65)
**6. Sudden Passion**					–	2.59 (0.98)

Legal judgment: 0 = Second-degree murder, 1 = First-degree murder.

* *p* < .05.

** *p* < .01.

Second, did aspect manipulations affect perceptions of the presence of temporal dynamics and the definitional dimensions? A series of independent samples *t*-tests compared the imperfective murder condition to the perfective murder condition (see Table 7). Findings do not indicate that the manipulation of aspect affected judgments of intentionality, seriousness of the provocation, self-defense, or sudden passion. Again, there was no support for the intentionality hypothesis. In contrast, as with Experiments 2 and 3, there was support for the temporal perspective hypothesis. The number of murder iterations was significantly higher in the imperfective condition than the perfective condition. As such, all three experiments that assessed temporality demonstrate that aspect had robust and consistent effects on the understanding of the temporal dynamics of the scenarios.

**Table 7:** Experiment 4 Group Means and Independent Sample T-Tests.

	Imperfective Murder		Perfective Murder		*t*-test Results
Variable	*M*	*SD*	*M*	*SD*	*t* (Cohen’s *d*)
Intentionality (Murderer)	3.09	0.85	2.95	0.97	0.88 (0.15)
Iterations (Murderer)	5.44	4.95	1.38	1.11	6.98 (1.13)***
Serious Provocation	2.15	0.90	2.21	0.99	-0.39 (-0.06)
Self-Defense	1.29	0.64	1.25	0.67	0.39 (0.06)
Sudden Passion	2.61	1.05	2.57	0.94	0.25 (0.04)

Df for each t-test is 137.

*** *p* < .001.

Third, did aspect manipulations affect legal judgments? Using a similar binary logistic regression analysis as the previous experiments (perfective murder = 1, imperfective murder = 0), the full model was statistically non-significant, χ^2^ = 0.281, *p* = .596, *Nagelkerke’s R* ^*2*^ = .003. Thus, the predictor (murder aspect) was not statistically significant.

Fourth, what are the relationships between the aspect manipulation, temporal dynamics, and legal judgments? A mediational analysis found that the murder aspect did not predict first-degree murder convictions indirectly through perceived number of murder actions. Specifically, when murder is presented in the imperfective (vs. perfective), participants perceived more murder actions (*a* = -4.06, *p* < .001); however, murder actions did not predict the likelihood of a first-degree conviction (*b* = 0.10, *p* = .170). The indirect link between the aspectual category of murder and legal judgments was not significant (*ab* = -0.37; 95% bias-corrected bootstrap CI [-1.03 to 0.11]), nor was the direct link (*c*’ = 0.18, *p* = .673).

In sum, the grammatical aspect of the murder verb appeared to influence the perception of the number of murder actions but not perceptions of intentionality or legal judgments. When compared against the first three experiments, these null findings demonstrate that the hypothesized mechanisms of emphasis associated with aspect (perceived temporality and intentionality) may not always result in meaningful differences in how situation models are constructed and evaluated. A minimally provoked (vs. highly provoked) murder may be viewed as inherently more culpable, which is evidenced by comparing the percentage of first-degree judgments of the first three experiments (25.5%, *N* = 560) against that of Experiment 4 (54.0%, *N* = 139). However, investigating interactive effects between semantic contexts (e.g., minimally provoked murder vs. highly provoked murder) and aspect manipulations were not possible given the design of Experiment 4.

Lastly, the results of Experiment 4 are inconsistent with a similarly designed experiment conducted by Hart and Albarracín [1]. Based on the failure of our series of experiments to consistently replicate Hart and Albarracín’s [1] finding that the imperfective aspect enhances perceived intentionality, we initiated a *Perspectives on Psychological Science* registered replication project of the Hart and Albarracín experiment [35]. A meta-analysis across 11 lab experiments showed no effect of aspect on intentionality and neither did a large-scale online replication also included in the article. This suggests that Hart and Albarracín’s [1] findings were a false positive [35]. Thus, differences between Experiment 4 and Hart and Albarracín [1] are likely due to spurious findings by Hart and Albarracín and not necessarily semantic differences between each study’s vignettes (e.g., violence resulting in death versus violence resulting in spinal cord injury). Still, future research is needed to investigate how grammatical aspect can potentially interact with various features of linguistic input. In addition to systems of emphasis not related to aspect (e.g., order of mention), as demonstrated in Experiment 3, semantic context may present a boundary condition for the impact of aspect on complex cognition.

## General Discussion

Would choosing “hitting” over “hit” when describing the actions of a murder influence the jury when making legal judgments? Would it similarly matter if the actions of a provocateur were described with an imperfective rather than a perfective aspect? This study addressed these questions and, in particular, if manipulations of grammatical aspect affected how a legal definition was applied to a specific scenario. The four experiments demonstrate that aspect may influence the construction and subsequent evaluation of situation models within the context of legal decision-making. However, the current results also demonstrate that these effects are dependent on other linguistic factors of the scenario. To illustrate, Experiments 1 and 2 found evidence that provocation in the imperfective (“was punching”) led to a lower likelihood of first-degree murder judgments when compared to provocation in the perfective (“punched”). However, in Experiment 3, this effect was not manifested when the murder action was mentioned first and the provocation second, suggesting that the effect is subject to ordering effects [33, 34].

Similar to Hart and Albarracín’s study, Experiments 2 and 3 demonstrated that the aspect of a given action is associated with the attribution of intentionality to the given verb [1]. But what is the nature of this association? Experiment 1 found no support for a link between aspect and intentionality and Experiments 2 through 4 found much stronger effects of aspect on perceived temporality than perceived intentionality. Therefore, aspect may not function as a reliable prime for semantically abstract information such as intentionality. In comparison to intentionality, temporal information is more semantically connected to the source word and, therefore, should be more strongly primed (for a similar argument, see [36]). Thus, aspect seems to directly prime temporal dynamics that can, in turn, influence more abstract and distal processing (e.g., committing attributions of intentionality and culpability). Indeed, the serial multiple mediational analysis of Experiment 3 demonstrates that perceiving higher iterations of violent actions results in greater perceived intentionality. All together, the current results indicate the link between aspect and perceived intentionality is both indirect and unstable.

An important difference between the present study and Hart and Albarracín’s study [1] is that participants were not only asked to rate intentionality but also to apply a very specific legal definition of first-degree murder to the murder scenario. We sought to understand if and how aspect may directly or indirectly influence conscious decision-making, namely in a legal context. Across all four experiments, consistent evidence indicated that participants applied the legal definition to their decisions. Intentionality judgments were positively correlated with first-degree murder judgments, whereas judgments regarding seriousness of provocation, self-defense, and sudden passion were appropriately negatively correlated with first-degree judgments. We found evidence that aspect can affect some, though not all, of these judgments. Importantly, mediational analyses suggest that impact of the aspect manipulations on first-degree murder judgments were mediated by the perception of different dimensions specified by the legal definition. This mediating effect was most consistently manifested between the murder aspect and perceptions of intentionality (Experiments 2 and 3). Again, while this effect is consistent with Hart and Albarracín, the failure to replicate that study in Experiment 4 must temper conclusions regarding a direct link between aspect and intentionality. As indicated by the serial multiple mediational analysis conducted for Experiment 3, support for the intentionality hypothesis may be a byproduct of the influence of the imperfective aspect on temporal dynamics. At this juncture, the current findings and extant literature suggest the influence of aspect on decision-making operates chiefly through its primary effect on time course and completion status.

There is a considerable amount of theoretical speculation [12–14, 37] and empirical research demonstrating that grammatical aspect affects various aspects of situation model construction [9, 10, 17–19]. Consistent with this research, Experiments 2 through 4 showed relatively robust evidence for the temporal perspective hypothesis. That is, murder and provocation actions were perceived to be more iterative (and therefore more durative) when conveyed with an imperfective aspect than with a perfective aspect. While there are a few studies that show that manipulations of aspect can affect complex reasoning and problem solving (e.g., 26]), the effect sizes for these effects (including the present study) are relatively small. Moreover, the inconsistent effects of aspect on the legal judgments in the context of the present study (either direct or indirect) suggest that the influence of aspect on complex, higher order cognition may be limited to situation model construction.

One can confidently conclude that grammatical aspect influenced how temporal features of a legal scenario were perceived in a direct, robust manner, and therefore likely affected how the events were represented in a situation model (i.e., the murder or provocation actions were iterative or a single event). Indeed, it has been speculated that grammatical morphemes, such as aspect, serve as processing instructions for situation model construction [8, 37]. While it is well known that the nature of situation models can influence reasoning and problem solving [38], the present study found inconsistent evidence that temporal judgments were directly correlated with legal decision-making. However, the legal definition did not emphasize temporality as a dimension to consider when deciding if a first-degree murder decision was warranted. It is reasonable to predict that if we artificially emphasized temporal dynamics in the legal definition, then such effects would have emerged. Future studies should further investigate how aspect influences legal reasoning indirectly through situation model construction.

Future researchers should bear in mind several limitations to the generalizability of the present study’s results. Most importantly, all four experiments used variations of the same vignette, which itself was informed by previous research (i.e., [1]). The observed effects cannot be easily disconnected from the vignette’s precise situation and characters. Thus, research is needed to examine described effects in broader contexts. Similarly, future research is needed on a broader range of verbs with respect to perceived intentionality and legal decision-making, particularly verbs that vary on the punctual-durative semantic dimension, as the imperfective aspect may not temporally extend verbs that must convey duration (e.g., strangle; [13]). To further assess the importance of duration on perceived intentionality, future studies can explicitly manipulate described durations (e.g., “strangled for 30 seconds” vs. “strangled for 60 seconds) and assess any additional impact of aspect manipulations (e.g., strangled vs. strangling). Lastly, alternative explanations may still exist for the effects of aspect described in the present study and previous research. For example, vignettes in this line of research, including the present study, often mix perfective and imperfective verbs within the same vignette, which may be experienced as odd or disfluent and plausibly impact the reader in unmeasured ways such as feeling uncertain or perhaps even reading slower. The inability to rule-out these possibilities is a limitation of the present study.

In conclusion, the findings reported here support the idea that legal decisions can be affected by grammatical aspect but the most robust effects were limited to temporal dynamics. Grammatical aspect has indirect influences on legal judgments to the extent that variability in aspect changes the features of a situation model that align with criteria for making legal judgments (e.g., legal definitions). One implication of this line of research is that persuaders (e.g., lawyers) may meaningfully alter mental representations of decision-makers (e.g., jurors) by using the imperfective aspect to selectively and subtly emphasize advantageous information. However, the extent to which an aspectual category meaningfully influences situation model construction and evaluation seems to be dependent upon the larger linguistic and semantic context. Grammatical morphemes are but just one factor in a complex decision process.
